# A Genetic Map of the Modern Urban Society of Amsterdam

**DOI:** 10.3389/fgene.2021.727269

**Published:** 2021-11-30

**Authors:** Bart Ferwerda, Abdel Abdellaoui, Max Nieuwdorp, Koos Zwinderman

**Affiliations:** ^1^ Department of Clinical Epidemiology and Biostatistics, Amsterdam University Medical Centers, Amsterdam, Netherlands; ^2^ Department of Psychiatry, Amsterdam UMC, University of Amsterdam, Amsterdam, Netherlands; ^3^ Department of Vascular Medicine, Amsterdam University Medical Center, Amsterdam, Netherlands; ^4^ Internal Medicine, Amsterdam University Medical Center, Amsterdam, Netherlands; ^5^ Institute for Cardiovascular Research, Amsterdam University Medical Center, Amsterdam, Netherlands

**Keywords:** HELIUS, multiethnic cohort, admixture, genetics, GWAS

## Abstract

Genetic differences between individuals underlie susceptibility to many diseases. Genome-wide association studies (GWAS) have discovered many susceptibility genes but were often limited to cohorts of predominantly European ancestry. Genetic diversity between individuals due to different ancestries and evolutionary histories shows that this approach has limitations. In order to gain a better understanding of the associated genetic variation, we need a more global genomics approach including a greater diversity. Here, we introduce the Healthy Life in an Urban Setting (HELIUS) cohort. The HELIUS cohort consists of participants living in Amsterdam, with a level of diversity that reflects the Dutch colonial and recent migration past. The current study includes 10,283 participants with genetic data available from seven groups of inhabitants, namely, Dutch, African Surinamese, South-Asian Surinamese, Turkish, Moroccan, Ghanaian, and Javanese Surinamese. First, we describe the genetic variation and admixture within the HELIUS cohort. Second, we show the challenges during imputation when having a genetically diverse cohort. Third, we conduct a body mass index (BMI) and height GWAS where we investigate the effects of a joint analysis of the entire cohort and a meta-analysis approach for the different subgroups. Finally, we construct polygenic scores for BMI and height and compare their predictive power across the different ethnic groups. Overall, we give a comprehensive overview of a genetically diverse cohort from Amsterdam. Our study emphasizes the importance of a less biased and more realistic representation of urban populations for mapping genetic associations with complex traits and disease risk for all.

## Introduction

The city of Amsterdam is a modern urban society with a vibrant composition of different ethnicities. It is one of the most diverse cities in Europe with ethnic minorities making up more than half of its population. The history of the Netherlands is well represented in the diversity of the communities that live there. Several communities are from the Dutch former colony the Republic of Suriname. During the colonial period, plantation labor was carried out by African slaves and after the abolition of slavery by indentured laborers from Asia. The history of forced or voluntary migration of ethnic groups from diverse continents is reflected in the current Surinamese population. Three of the main Surinamese ethnicities living in Amsterdam are the Afro-Caribbean, hereafter named African Surinamese; the South-Asian; and the Javanese Surinamese. Besides Surinamese, there are Moroccan, Turkish, and Ghanaian communities from recent migrations living in Amsterdam. This diversity has consequences for the city’s health-care system because the ethnic background can be associated with disease susceptibility, progression, and medication response (Wilson et al., 2001; Gurdasani et al., 2019). Differences between ethnic groups are partly based on cultural differences, differences in lifestyle, socioeconomic factors, and genetic ancestry, which may result partly from different evolutionary histories. For example, research showed that the effect of hypertension treatment with ACE inhibitors is less effective than calcium blockers for patients with African ancestry (Brewster and Seedat, 2013). For treatment of chronic hepatitis C virus infection, it is found that the treatments are less effective for African Americans due to genetic polymorphisms (Ge et al., 2009). These studies show that European-based results regarding effective disease treatments cannot always be generalized to individuals of non-European ethnicities. This bias is also seen in much genetic research that aims to understand the susceptibility of diseases.

The genome-wide association study (GWAS) approach has proven to be successful in identifying genomic regions associated with diseases and in studying how and to what extent genetic variation contributes to disease susceptibility (Visscher et al., 2017; Tam et al., 2019; Claussnitzer et al., 2020). In the early GWAS, the focus was mainly on homogenous European cohorts, but lately, there has been a shift toward a more trans-ethnic strategy (Vujkovic et al., 2020). Despite the shift toward more ethnically diverse cohorts, the diversity is still skewed in current published data with ± 7% Asian, ± 1% African American or the Caribbean, and less than 0.4% African data compared to ± 92% European (Mills and Rahal, 2020). This imbalance in ancestry forms the basis of results that are difficult to translate from Europeans to populations with other ethnicities, as illustrated, for example, by the Eurocentric bias in the predictive power of polygenic risk scores (PRSs). A PRS predicts an individual’s risk for a specific disease based on how many risk alleles he/she carries (Wray et al., 2007). These risk scores are mostly based on European GWAS results and perform notably worse when applied to cohorts with other ancestral origins (Martin et al., 2019). Differences in disease risks, susceptibility, treatment, and the appliance of genomic methods due to ethnic background are still underrepresented in genomics research, and a shift toward a more inclusive global approach is needed (Claussnitzer et al., 2020).

An important step toward global genomics is to compose cohorts from the modern ethnic urban landscapes and a trans-ethnic inclusion of participants. This more diverse composition of cohort sampling likely ensures a more representative genomic disease prediction and optimized treatment decisions. The Healthy Life in an Urban Setting (HELIUS) study was initiated to address multiethnic representation in life science research. HELIUS is a prospective cohort study characterized by several large ethnic groups living in Amsterdam (Snijder et al., 2017). A cross-selection of 10,283 HELIUS participants was genotyped to get insights into the genetics of this cohort. Here, we first present a population genetic overview showing the diverse genetic composition of the HELIUS cohort. The population genetics show complex genetic diversity between and within the HELIUS ethnic groups. Second, we investigated the best quality control and genotype imputation strategies of this trans-ethnic cohort. Third, GWASs on body mass index (BMI) and height were performed to compare results of the individual ancestral groups with a cross-ancestry meta-analysis. Finally, PRSs were constructed for BMI and height based on large European GWASs, and their predictive power was assessed across all ethnic groups. Overall, this study provides an overview of challenges and difficulties of genetic analyses when using ethnically diverse cohorts and highlights the importance of this broader genomics approach.

## Materials and Methods

### HELIUS Cohort

Healthy Life in an Urban Setting (HELIUS) is a prospective cohort study executed in Amsterdam, characterized by ethnic diversity (Martin et al., 2019). HELIUS includes six large groups of inhabitants of Amsterdam, namely, Dutch, African Surinamese, South-Asian Surinamese, Turkish, Moroccan, or Ghanaian background, and one small group with a Javanese Surinamese background (Stronks et al., 2013). The HELIUS cohort consists of approximately 25,000 participants aged 18–70 years. For most participants, data on social, environmental, and biological determinants were collected, and follow-up data are obtained. Detailed information on the cohort participants and gathered data has previously been published (Stronks et al., 2013; Snijder et al., 2017). In addition to the general measurements and questionnaires, specific measurements such as microbiomes were also collected for a cross section of the participants (Deschasaux et al., 2018). HELIUS was compiled according to the Declaration of Helsinki (6th, 7th revisions), and ethical approval from the Amsterdam University Medical Centre (location AMC) Medical Ethics Committee was obtained. All participants approved by giving written informed consent.

### DNA Isolation, Genotyping, and Quality Control

A cross-selection of 10,283 HELIUS participants was made for genotyping. Whole blood for DNA isolation was collected in EDTA tubes and stored at −80°C in the AMC Biobank. DNA was isolated using the Gentra Puregene Isolation Kit (Qiagen), and quality control procedures were performed to determine the DNA yield and purity.

DNA was shipped to the Erasmus MC Human Genomic Facility where genotyping was performed. For genotyping, the Illumina Global Screening Array 24v1-0 designed for the multiethnic genome-wide content purpose was used. An in-house protocol of the Human Genomic Facility, with Illumina’s GenomeStudio software, was used to perform the initial genotyping of the array. Subsequently, a second quality control (QC) was performed for removing the individuals with discordant gender information and when more than 5% called data on markers per individual were missing.

A general QC for the autosomal markers was executed removing variations with more than 5% calls missing, minor allele frequencies (MAF) of <1%, violation of the Hardy–Weinberg equilibrium (HWE) (*p* ≤ 10^−5^), and heterozygosity deviations from a mean larger than ± 3 SD (Anderson et al., 2010). Because the cohort consists of participants with different ancestries, where allele frequency differences between the groups can influence the QC, two different QCs have been performed. First QC was done on all samples together. Using PLINK, data were merged and pruned with the 1000G cohort to perform a principal components analysis (PCA), using smartpca, for determining the ancestry based on the genomic background (Patterson et al., 2006; Purcell et al., 2007; Genomes Project et al., 2015). HELIUS database–reported ethnicity and PCA were used to detect divergent genetic ancestries, which were removed. For the Surinamese participants, the database-reported ethnicity was retained because varying degrees of admixture make it difficult to strictly define clusters. All homogeneous clusters were thereafter subtracted from the data, and a second QC was specifically performed on all samples from the same ethnicity. This resulted in eight QCed datasets, namely, of all samples together, and of participants with African Surinamese, South-Asian Surinamese, Javanese Surinamese, Ghanaian, Moroccan, Turkish, and Dutch ancestries. All markers are reported with respect to the reference allele and coordinates of GRCh37.

### Genetic Ancestry and Admixture

For all population genetic analyses, a dataset was created where all QCed HELIUS samples were merged with 1000G cohort samples. After merging, variations on the genomic high LD regions were filtered out, and the remaining dataset was pruned using PLINK, as described in Anderson et al. (2010). Smartpca from the EIGENSOFT package was used for modeling ancestry differences between samples using a principal components analysis (PCA) approach (Patterson et al., 2006). Because it is known that the HELIUS dataset includes admixed individuals, namely, the Surinamese samples, the ancestry was estimated (Micheletti et al., 2020). Estimates of the degree of mixed ancestry were obtained using ADMIXTURE software (Alexander and Lange, 2011). ADMIXTURE estimates were run, starting with 2 up to 10 ancestral populations (K). Cross-validation within the ADMIXTURE package was used to infer the best fit.

### Imputation

To determine the imputation performance of different reference panels on the GSA array, a random set of 25% of the genotyped markers of the arrayed were removed per ethnic group. The random markers were generated for each ethnic group separately to minimalize any effect of population-specific markers. After marker removal, imputation was performed using the TopMed imputation panel and server, the Michigan imputation server, and the Sanger imputation server (Das et al., 2016; McCarthy et al., 2016; Kowalski et al., 2019). The TopMed and Michigan imputation servers use Eagle2 for phasing the data and Minimac4 for the imputation (Das et al., 2016). We used version R2 of the Trans-Omics for Precision Medicine (TopMEd) reference panel, which is built on a subset of 97,256 samples with a multiethnic background. With the Michigan imputation server, the ethnically diverse 1000G phase 3 panel consisting of 26 populations and the African American Panel (CAAPA) were used on the African Surinamese (Mathias et al., 2016). For phasing, the Sanger imputation server used Eagle2, but for imputation, it uses PBWT (Durbin, 2014; McCarthy et al., 2016). Reference panels used with the Sanger imputation server were the 1000G phase 3 panel and the Haplotype Reference Consortium (HRC) reference panel version 1.1 consisting of 32,470 samples of mostly pan-European and the 1000 Genomes Phase 3 (McCarthy et al., 2016).

After imputation, the removed markers per ethnicity were filtered out and compared with the array genotype calling results. Disagreements between imputations and measured genotypes per individual were determined for each imputation reference panel separately. As an indication of imputation accuracy, the percentage of mismatches per ethnicity is reported.

All imputations were performed on data after the QC of all samples together and repeated for all ethnicities separately. Marker disagreements between both imputation methods were determined after the QC of the imputations. Within the QC, all markers were removed with an MAF <1%, HWE *p* ≤ 10^−5^, or INFO scores <0.4, for Sanger imputations, or R2 <0.3 for Michigan and TopMed, or when a marker was only imputed by one of the imputation methods. After QC, marker disagreement was determined and expressed as percentage.

### GWAS and Meta-Analysis

TopMed and Sanger 1000G imputations were used for the subsequent GWAS analysis. It was decided to make the filtering stricter for the Sanger 1000G imputation that performed less during the testing of the imputation performance. For the GWAS, all markers were removed with an MAF <1% or *R* ^2^ 0.03 (TopMed) or INFO scores <0.8 (1000G Sanger imputation). Height phenotypes were measured as individual’s length in centimeters. For the height analysis, gender, age (Wilson et al., 2001), and the first 10 PCs were included in the analysis as covariates. BMI was regressed on age (Wilson et al., 2001) and the first 10 PCs of the genetic data to obtain residuals. This was done separately by sex and inverse-normally transformed to obtain a normal distribution. The procedure was performed on the entire cohort and for all ethnicities separately when conducting the analysis used for the meta-analysis.

Sample relatedness and population stratification in diverse cohorts, like HELIUS, are confounding factors that could lead to spurious associations. Besides the inclusion of PCs as covariates to further control for these confounding factors, the mixed linear model (MLM)–based tool fastGWA was used for the GWAS (Jiang et al., 2019). Height and BMI associations were calculated for the entire cohort, namely, the joint analysis, and for each ethnicity separately. Due to the small number of genotyped Javanese Surinamese, they were excluded in the ethnicity-specific GWAS and meta-analysis.

All GWAS ethnic-specific analyses were used to determine the best meta-analysis. For determining the best meta-analysis method, several were first applied on the BMI results, namely, the fixed-effects (FE), random-effects (SE), and the Han and Eskin’s random effects model (SE2) using Metasoft (Han and Eskin, 2011). Beside these three methods, two specific trans-ethnic meta-analysis methods were also used, namely, MANTRA and MR-MEGA (Morris, 2011; Mägi et al., 2017). Most of the methods showed similar associations for markers, with a *p*-value ≤1.0e-5 (Supplementary Figure S5). The specific trans-ethnic MR-MEGA attempts to correct for the genetic variation between the ethnic groups as covariates and was therefore chosen for comparison with the results of the entire cohort analysis for BMI and height.

### Heritability and PRS

Heritability estimation for height and BMI was calculated using GCTA-GREML (Yang et al., 2015). In summary, the segment-based LD scores were first calculated for the entire cohort and all ethnicities separately using the unimputed QCed genotypes. LD scores were used to stratify the SNPs in quarters and used to calculate the genetic relationship matrices (GRMs). GRMs were used in mixed-model regression analyses using restricted maximum likelihood for the heritability estimation.

Polygenic risk scores were calculated using the protocol described in Choi et al. (2020). In summary, the GWAS statistics for height and BMI were used from Yengo et al. (2018). The obtained GWAS summary statistics were checked on genome built and filtered on SNPs with an MAF ≥1%. All duplicates and ambiguous results were removed from the summary statistics. For HELIUS, the imputed genetic data of all ethnicity SNPs with an MAF ≥1%, HWE ≤1e-6, ambiguities, and duplications were excluded. To prevent mismatching of variations between files, all were checked on strand-flipping and recoded to match the GWAS summary statistics.

PLINK was used for clumping and calculating the polygenic scores with the score function. PRS was calculated for *p*-value thresholds 0.001, 0.05, 0.1, 0.2, 0.3, 0.4, and 0.5. Regression in R, with correction for the first 10 PCs, was used to determine the “best-fit” PRS for each ethnicity, explaining the highest phenotypic variance. Bootstrapping was used to calculate the CI for each “best-fit.” Results were plotted with the European ancestry proportion based on the highest K in European ethnicities of the ADMIXTURE K = 3 prediction.

## Results

### Determination of the Genetic Background Within the HELIUS Cohort

**FIGURE 1:**
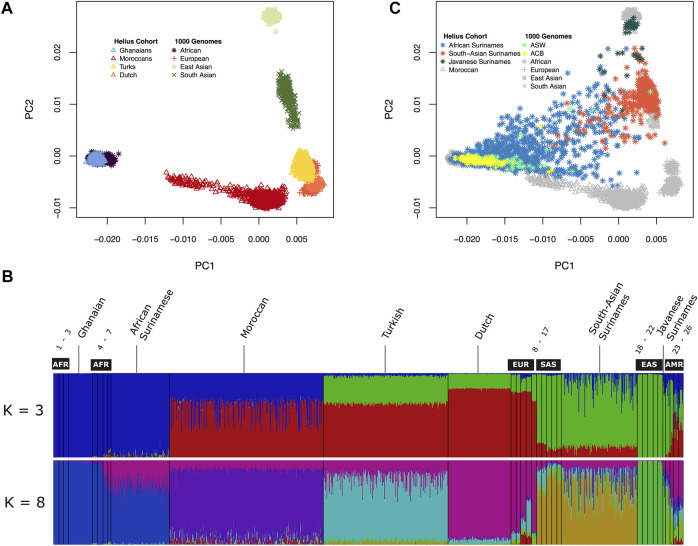
Genetic diversity between the HELIUS Cohort populations. Ancestry was explored by merging the HELIUS cohort together with the 1000 Genome project populations. Differences were first inferred by using principal components analysis (PCA). **(A)** First two PCAs of the non-admixed populations grouped at their continental origin. Population ancestry was estimated using ADMIXTURE. **(B)** Results of a genome-wide ancestry with K = 3 or 8 ancestral populations. All HELIUS populations are displayed, and 1000G populations are marked by their continental origin (AFR = African, EUR = European, SAS = South Asian, EAS = East Asian, and AMR = Admixed America). Numbers indicate the exact 1000G populations which can be found in Supplementary Figure S3. PCA of the Surinamese HELIUS participants in **(C)** together with the African American (ASW) and African Caribbean (ACB) admixed 1000G populations highlighted.

The multi-ancestral Global Screening Array (GSA) was used for genotyping the HELIUS cohort (Supplementary Figure S1). After genotyping and quality control (see *Materials and Methods* section), principal component analysis (PCA) of the genotypes was used to check congruence between genetic ancestry and the HELIUS database reported ethnicity. PCA was performed after merging the HELIUS cohort with the 1000 Genomes (1000G) cohort to evaluate the clustering with known reference populations (Genomes Project et al., 2015). Based on the first two principal components, a clear separation of the African, European, and Asian continental populations was observed when the admixed Surinamese were excluded (Figure 1A). All cohorts showed a clear clustering according to their ancestral origin.

### Admixture Within HELIUS

Because of the admixture within the different ancestries from the diverse continents within the Surinamese, we used the program ADMIXTURE to detect the substructure within the three Surinamese ethnic groups. The HELIUS cohort was merged with the 1000G cohort to infer these substructures and ancestral admixture. For the admixture method, it was necessary to make assumptions on the number of ancestral source populations (Supplementary Figure S3). Assuming three source populations (K = 3), admixture showed the amount of African, Asian, and European ancestries within each individual but did not show the similarities between the HELIUS cohort ethnicities very well (Figure 1B). In contrast, the best inference by cross-validation K = 8 provided more insights into the admixture within the ethnicities (Figure1B, Supplementary Figure S3). Admixture showed that South-Asian Surinamese have a comparable ancestral pattern as the 1000G populations from South Asia, but some individuals do seem to have a larger amount of African ancestry. This African ancestral admixture is also seen when displaying the Surinamese in the PCA plot (Figure 1C). Another observation was that the scattered clustering seen within the African Surinamese was larger than that in African Americans (Levene’s test *p*-value = 0.002 for C1 and *p*-value = 0.003 for C2) and other African Caribbean’s (Levene’s test *p*-value = 9.2e-13 for C1 and *p*-value = 2.0e-08 for C2), possibly indicating different degrees of ancestry variance. The genetic background of the HELIUS cohort demonstrates the complexity of the diversity in individuals with widely separated geographic ancestry and admixture.

### Imputing the HELIUS Cohort

**FIGURE 2:**
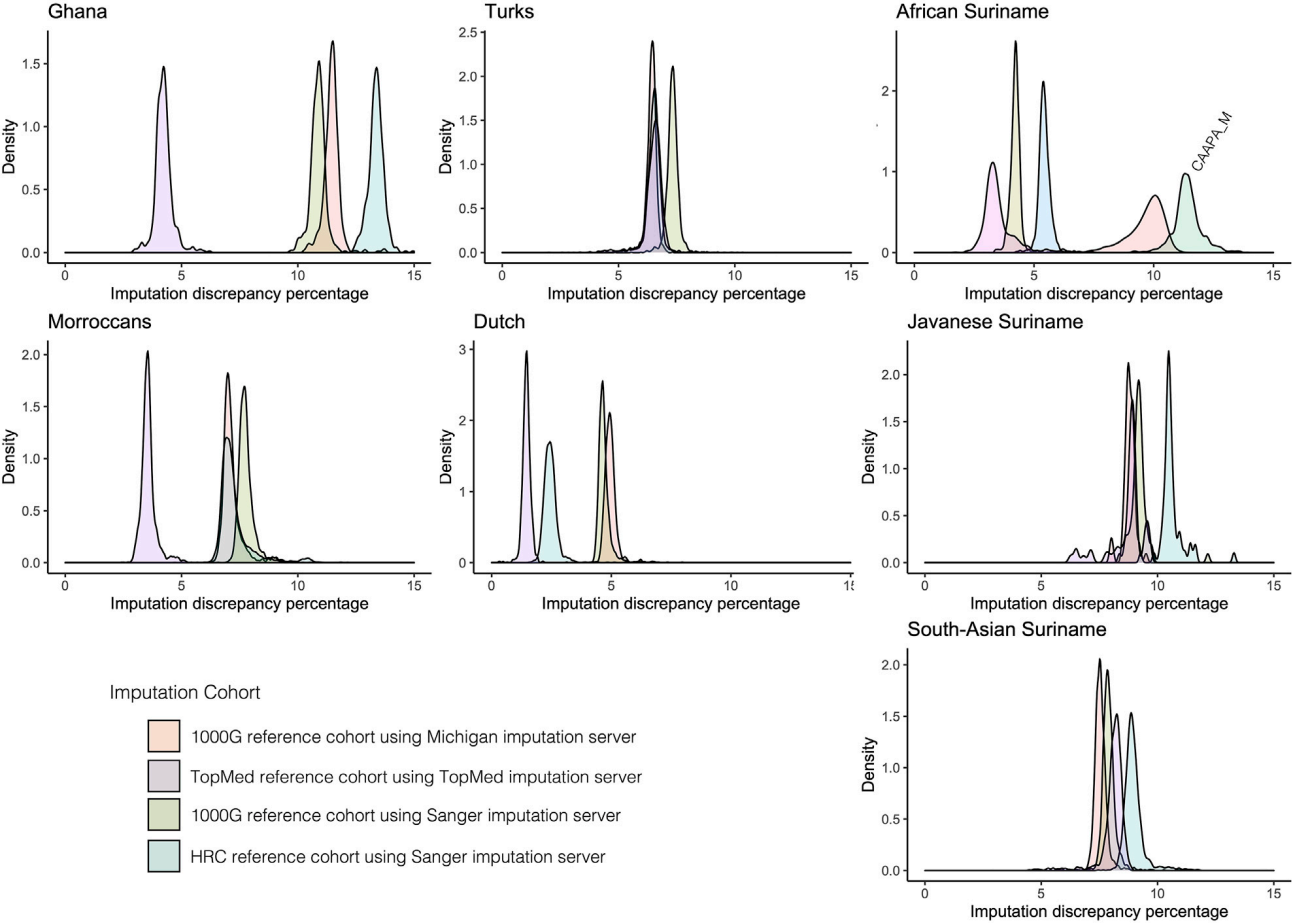
Comparison of imputation reference panels on HELIUS. Imputation performance was determined by filtering out 25% of the measured markers per ethnicity. After filtering out, the markers imputation was performed with the Sanger server using the 1000G and HRC imputation reference panels, Michigan imputation server with the 1000G, and TopMed imputation reference panel. The Michigan imputation cohort CAAPA was also used for imputation on the African Surinamese. Plots show the density of the percentage mismatches between imputed and measured genotypes per individual for each imputation server and reference panel per ethnicity.

Imputation reliability is highly dependent on the use of the correct reference populations containing adequate haplotype diversity compared to the imputed cohort. Imputation reference panels from TopMed, Michigan, and Sanger imputation servers using removed GSA array markers for the HELIUS ethnicities were used to get an indication of imputation accuracy. The Sanger imputation server was used with the 1000G and Haplotype reference panel (HRC) (McCarthy et al., 2016). For the Michigan imputation server, 1000G and TopMEd were used, and the African American reference cohort (CAAPA) was evaluated only for the African Surinamese (Vergara et al., 2018; Kowalski et al., 2019). After imputation, the percentages of mismatches were determined for each ethnic group as a degree of imputation discrepancy, as shown in Figure 2. In general, the discrepancy between the imputation methods and used reference panels for each ethnicity showed no large differences. Differences between the ethnicities showed that the large TopMed reference panel including a large number of African Americans performed best within the Ghanaian, Moroccan, Dutch, and African Surinamese ethnicities. This highlights the importance that the reference panel and included haplotypes closely capture the diversity of the cohort.

### Genome-Wide Association Study on Separate Ethnic Groups vs. Meta-Analysis

Population stratification can lead to false-positive or false-negative signals in a genome-wide association study when samples with different ancestries are analyzed simultaneously. Some methods have been developed that take population structure and relationships between samples into account, such as linear mixed-effects regression models (LMM) (Yu et al., 2006). GWAS datasets with varying ethnicities can be analyzed in two ways, namely, by analyzing the entire cohort at once or by meta-analysis summarizing the results of analyses per ethnicity (Peterson et al., 2019; Wojcik et al., 2019). To investigate outcome differences between the two approaches, we applied a GWAS on height and BMI where height is likely less sensitive to environmental factors (Zhou and Lee, 2021). The associations for height and BMI were investigated using a linear mixed-model GWAS applied to the entire HELIUS cohort and also per ethnicity. For the meta-analysis, performance of several methods has been compared (Supplementary Figure S4). Eventually, the multiethnic method MR-MEGA, which takes the genome-wide diversity of the included populations into account, was chosen for the comparisons (Mägi et al., 2017). For evaluating the effect of the imputation method, we chose the TopMed and Sanger imputation server results. Table 1 gives an overview of the genotyped cohort and imputation numbers.

**TABLE 1:** HELIUS study cohort overview of genotyped participants with number of markers genotyped and imputed.

Population/ethnicity	N	Age (years)	Gender (% female)	Array markers^b^	TopMed^c^ imputation	Sanger 1000G^d^ imputation
Ghanaians	480	48.0 ± 9.2	57.3	352,956	14,170,590	11,684,750
Moroccans	3048	40.8 ± 12.8	60.6	436,920	10,314,717	9,216,391
Turks	2649	40.6 ± 12.1	53,6	458,667	8,226,331	6,674,703
Dutch	1287	51.8 ± 12.6	50.3	487,008	8,257,774	8,436,030
African Surinamese	1156	51.7 ± 10.5	60.6	381,499	14,011,528	12,324,000
South-Asian Surinamese	1502	46.9 ± 13.2	53.9	406,867	8,136,254	7,973,372
Javanese Surinamese	57	51.1 ± 10.9	52.6	373,195	7,575,865	7,213,944
HELIUS (joint)	10283^a^	44.7 ± 13.1	56.2	327,690	10,427,937	7,001,772

a A total of 104 samples come from other than these 7 backgrounds.

b Illumina GSA array resulted in 700078 genotyped markers before QC. For each population, QC included the markers present in at least 95% of the individuals, MAF below 1%, and deviation of Hardy–Weinberg equilibrium (*p* ≥ 0.00001).

c TopMed imputed markers were filtered on SNPs only, MAF ≥1%, and *R* ^2^ ≥ 0.3.

d Sanger 1000G imputed markers were filtered on SNPs only, MAF ≥0.8, and INFO ≥0.8.

**FIGURE 3:**
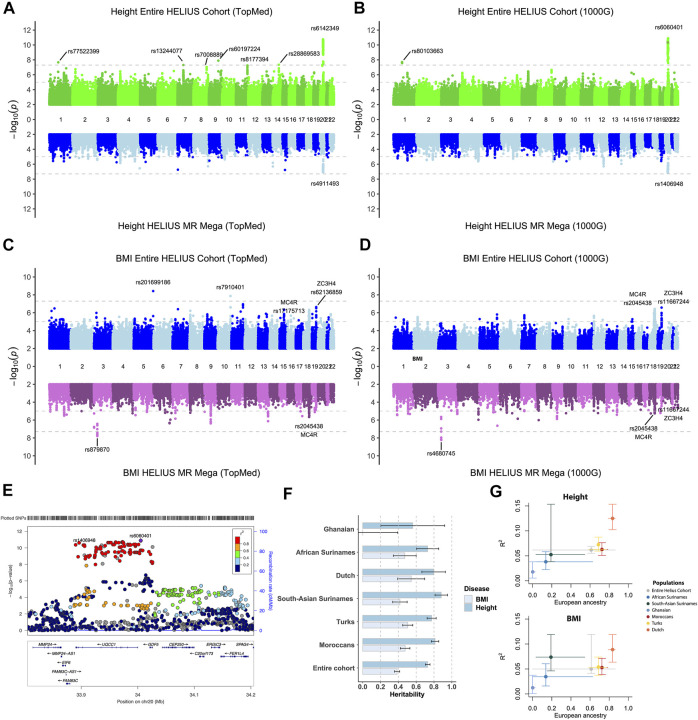
Height and BMI association study on the HELIUS cohort. Miami plot of the height and BMI using the TopMed **(A, C)** or Sanger 1000G imputation reference cohort **(B, D)** GWAS. Results of the entire cohort are plotted at the top panels, and the MR-MEGA meta-analysis combines the ethnic-specific GWAS results at the bottom panel. *Y*-axis displays the -log10 *p* values and the *x*-axis the associated marker location on each chromosome. Gray dashed lines indicate the 1.0e-5 and genome-wide significant 5.0e-8 *p*-value thresholds. **(E)** Locus zoom of the height association peak on chromosome 20 showing the associated loci UQCC1/GDF5 within the area. **(F)** Heritability, and standard error, bar plot for the entire or ethnic-specific cohort, and each ethnic-specific GWAS for the BMI and height. The PRS for BMI and height is displayed in **(G)**. The *y*-axis represents the “best fitted” R2 for the entire cohort and each ethnicity with vertical error bars representing the bootstrapping confidence interval. The average proportion of European ancestry, based on the ADMIXTURE K = 3 calculations, for the entire cohort and per ethnicity is displayed at the *x*-axis. Horizontal lines were drawn between the smallest and largest measured European ancestry values for that group.

The entire cohort and ethnic-specific height and BMI GWAS results were compared with the results of previously published GWAS on those traits (Yengo et al., 2018; Lango Allen et al., 2010; Locke et al., 2015; Ng et al., 2017; Wood et al., 2014). Due to the modest sample size of the cohort, we only focused on the associations, with a *p*-value of <5 × 10e^−8^. Height’s strongest association with both the entire cohort and ethnic-specific meta-analysis was the GDF5-BFZB locus on chromosome 20 known to be involved in the alterations in bone growth and development (Figures 3A,B,E) (Sanna et al., 2008). With the analyses of the entire cohort, another signal was found on chromosome 1 that disappeared when conducting the meta-analysis. This variant has previously not been reported to be associated with height, and because the signal was based on only imputed SNPs, it is difficult without any replication to determine if this is a true new signal. With the different imputations, all detected the signal on chromosomes 1 and 20. With the TopMed imputations, five other signals were detected when analyzing the entire cohort (Figure 3A). Three of these associations on chromosome 7 (*p*-value = 1.4e-06), 8 (*p*-value = 2.3e-07), and 11 (*p*-value = 3e-06) did not reach genome-wide significance with the Sanger 1000G imputation. The signals on chromosomes 9 and 14 were only found when using the TopMed imputations. For markers in these signals, a two-fold higher frequency was observed in African populations. In line with the higher frequency of these markers in African populations, we found that the TopMed signal of the chromosome 7 marker was within the IGFBP3/TNS3 genomic area previously reported in a height GWAS in individuals of African ancestry (Graff et al., 2021). For the other four signals, we found no previously reported height associations, and without replication, it is hard to determine their reliability.

For BMI it showed that particularly for the entire cohort analysis, despite not being genome-wide significant, the strongest associations were for known BMI-associated gene regions MC4R, SEC16B, and, to a lesser extent, the FTO gene (Figures 3C,D, Supplementary Tables S1–S4) (Dina et al., 2007; Scuteri et al., 2007; Locke et al., 2015; Ng et al., 2017). Two signals were only found using the TopMed reference panel for imputation. In accordance with some of the height-specific signals, the BMI-associated signals on chromosomes 5 and 10 were due to higher frequencies of these markers within either the Asian or African ethnicity. The strongest associations of the joint analysis, namely, the region on chromosomes 18 (MC4F) and 19 (ZC3H4), were less prominent in the MR-MEGA approach. In contrast, MR-MEGA showed a significant association in a region on chromosome 3 independent of the used imputation. To confirm these signals, another meta-analysis method that was specifically developed for multiethnic cohorts, namely, the Bayesian method MANTRA, was used (Morris, 2011). Comparing the results of MANTRA with MR-MEGA showed an overlap of several associations, including the previously found signal on chromosome 3 (Supplementary Figure S4A). It also shows that geographic continental differences of the allele frequency for signals with stronger differences may be the underlying reason for observed chromosome 3 and more single-variant signals (Supplementary Figure S4B).

### SNP-Based Heritability for BMI and Height

SNP-based heritability for BMI and height was determined for the entire cohort and all HELIUS ethnicities separately (Figure 3F). The SNP-based heritabilities of both traits were comparable across the ethnic groups (except perhaps for BMI in the Ghanaian group, the smallest group, for which we likely lacked the statistical power for this analysis).

### PRS for BMI and Height

PRS results for the genetic complex height and BMI phenotypes were substantially different between ethnicities in our cohort. Especially the European ancestry of the cohort was correlated with the degree of explained phenotype-variance by the PRS (Figure 3G). The lower explained phenotypic variance for the Ghanaian and African Surinamese for height (0.018/0.038) and BMI (0.012/0.035) compared to the Dutch (0.125/0.089) indicates a reduction in the predictive accuracy for these ethnicities.

## Discussion

The Amsterdam Urban genetic map shows the diverse composition of the city and the challenges of urban sampling. Besides the clustering of all participants with their ethnic substructure, substantial admixture can be observed (Figure 1). Notable substructures were observed in the Moroccan group with clusters of individuals representing a possible north and south Saharan gradient. With the inclusion of all African and Asian populations, the Turkish and Dutch participants seemed to partly overlap. When zooming in to a more detailed picture of this overlap, excluding the African and Asian populations these subgroups showed the HELIUS Turkish and Dutch participants at opposite sides of the first component in accordance with a geographical south to north gradient (Supplementary Figure S2A). In addition, a substructure was observed among the Turkish participants who likely reflect the reported clustering with Middle Easterners, and South Asians (Hodoglugil and Mahley, 2012). Inclusion of the Moroccans within the European PCA analysis showed the described gene flow from the Near East, Europe, and sub-Saharan geographical regions (Supplementary Figure S2B) (Henn et al., 2012). In general, PCA revealed that there was a clear clustering between Ghanaian, Moroccan, Turkish, and Dutch ethnicities, but it also showed that within ethnicities, such as the Turkish and Moroccan, additional subgroups were observed.

Within the genotyped HELIUS participants, a large proportion of individuals had Surinamese descent with three distinct ethnic groups, namely, the African Surinamese, South-Asian Surinamese, and Javanese Surinamese. African Surinamese descended from the transatlantic slave trade to the Americas having roots in Western Africa (Henn et al., 2012; Micheletti et al., 2020). South-Asian Surinamese ancestry originated from the Indian subcontinent coming to Suriname as indentured workers. The smallest genotyped group consisted of Javanese Surinamese having their origin from the island of Java in the former Dutch East Indies where ancestors were contracted to come as workers to Suriname. These historical backgrounds and the different ancestries from diverse continents within the Surinamese were observed in the admixture substructure within the three Surinamese ethnic groups (Figure 1B). Another observation was that scattered clustering seen within the African Surinamese was larger than that in African Americans and other African Caribbeans (Figure 1C). The admixture within the Surinamese and the genetic substructure within Turkish and Moroccan will influence the performance of imputation and genetic association studies.

The effect ethnicity has on the imputation results revealed that both the imputation server and specific imputation reference cohort have an impact on the results. Our re-imputation data suggest that a close match of the genetic background from the imported individuals with the reference panel was important. This was also emphasized by studies of human genetic variation that discover new common genetic variation through the inclusion of new populations (Bergström et al., 2020). It also underlines the importance of increasing diversity in reference panels toward a more global genomic approach at least as long as array genotyping of large cohorts is more cost-efficient than a whole-genome sequencing approach.

Besides imputation, another challenge is performing GWAS with ethnically diverse cohorts. Despite the fact that the HELIUS cohort is smaller in size than cohorts of previous height and BMI studies, few of the reported associations were replicated. For the strongest associations, no big differences between the used imputation reference panels were observed. GWAS using the TopMed reference panel did however found more genome-wide associations. Better imputation of the HELIUS cohort with the TopMed reference panel could be the explanation for this. When using the entire cohort, the risk is that ethnic-specific variations will be lost during QC and not analyzed. This can be overcome by splitting the cohort into ethnic groups on which the QC and association testing are performed. The disadvantage of splitting the cohort into ethnic groups for the meta-analysis is even smaller groups were created, which is likely the reason that the replicated associations were weaker (Figures 3A–D). Also ethnic-specific signals, such as the TopMed height signal on chromosome 7, were lost using a meta-analysis. Another challenge was shown by the BMI ethnic-specific meta-analysis introducing other signals. Due to unique ethnicities within HELIUS, it is hard to determine if these observed signals are a true association or an artifact. Overall, we think that with a cohort of the size of HELIUS, a meta-analysis would be too strict, and when using a representative imputation reference panel, joint individual–level GWAS approach is sufficient.

Height and BMI were specifically chosen as a trait because of their high heritability. Compared to BMI, height showed a higher degree of heritability across all groups. This higher heritability of height than BMI is in line with previous findings reporting the heritability of these complex traits (Wainschtein et al., 2019; Zhou and Lee, 2021). Estimated heritability for BMI in the Ghanaian group showed that one must be careful while interpreting heritability when calculated within a small number of individuals. Our PRS results showed what the effect is of the used summary statistic. To construct the PRS of BMI and height, we used the summary statistics from a GWAS study that was conducted in individuals with European ancestry (Yengo et al., 2018). When comparing the prediction accuracy of the PRS between the HELIUS ethnicities, the populations with more European ancestry showed higher prediction accuracy (Figure 3G). These observations also illustrate the need for more non-European cohorts to better predict within multiethnic groups.

Altogether, the HELIUS cohort with its Amsterdam urban roots reflects the current genetic diversity of the Dutch metropolitan city. The uniqueness of the dataset lies in the considerable genetic diversity of the participants that all live in close geographic proximity with each other and that therefore all are exposed to similar urban environmental influences. Its diverse composition covers a substantial portion of the human population genomic diversity of today. The consequence of the genetic diversity and admixture within the population will be that the analytic methods must be carefully considered. An important point is the choice of the imputation reference cohort. With the genetic diversity of the HELIUS population, the most diverse reference panel is preferred. When comparing the BMI and height associations of the HELIUS cohort with the ethnic-specific meta-analysis, the entire cohort analysis showed stronger associations signals. The effectiveness of polygenic risk prediction is dependent on the ancestral background of the discovery GWAS, confirming the need for large-scale non-European GWAS efforts for a wider range of complex traits and diseases, to which the HELIUS cohort can potentially contribute. Shifting toward more diverse cohorts in genetic research has the potential to help health research improve for larger portions of our increasingly globalizing and ethnically diverse societies.

## Data Availability

The datasets presented in this study can be found in online repositories. The names of the repository/repositories and accession number(s) can be found below: www.heliusstudy.nl.
