# Supplementary material for: A Genetic Map of the Modern Urban Society of Amsterdam
Source: Front Genet. 2021 Nov 30;12:727269. doi: 10.3389/fgene.2021.727269 (PMC8670378; doi:10.3389/fgene.2021.727269)
Supplement: Supplementary file 1 [file DataSheet1.PDF]

Supplemental Data

Table S1*
Previously known GWS BMI loci in European meta-analysis presented in "Genetic studies of body mass index yield new insights for obesity biology (1)" extended data table 2

Table S2*

Novel GWS BMI loci in European meta-analysis presented in "Genetic studies of body mass index yield new insights for obesity biology (1)" extended data table 2

Table S3*

Novel and previously identified BMI markers from "Discovery and fine-mapping of adiposity loci using high density imputation of genome-wide association studies in individuals of African ancestry: African Ancestry Anthropometry Genetics Consortium" (2).

Table S4*

Meta-analysis results from GWAS tableS1.

*All tables can be found in the accompanied excel file

Figure S1 Marker distribution and minor allele frequency of the used GSA array.

A). Variation density plot of the +/- 678,424 Illumina GSA genotyped markers at each chromosome before QC. Grey areas on each chromosome are the centromeres. Chromosome six shows a region with high density tagging corresponding the HLA region. Plot was generated with the CMplot R package (Yin, L. et al. rMVP: A Memory-efficient, Visualization-enhanced, and Parallel-accelerated tool for Genome-Wide Association Study. bioRxiv, 2020.08.20.258491 (2020)).

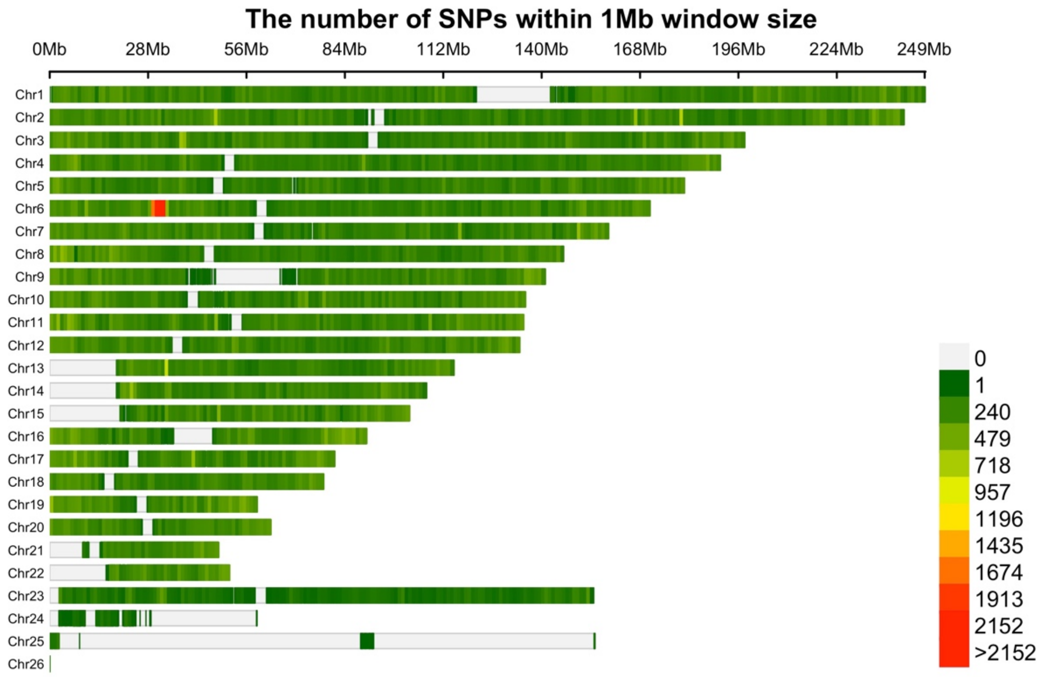

B). Minor Allele Frequency (MAF) distribution of the Illumina GSA genotyped markers before QC. X axis shows bins, set on 0.01, of the markers MAF and the y axis the frequency of markers in each bin. Distribution shows many markers with a relative low frequency. The inset zooms in on the lowest MAF markers distribution containing 50% of the genotyped markers where more than half fall within the lowest MAF bin.

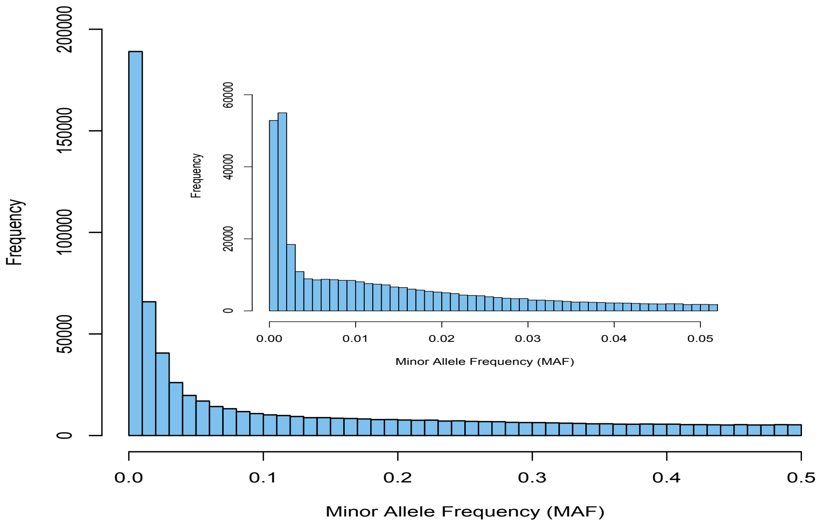

Figure S2

Multidimensional scaling (MDS) to visualize substructure and indices of population genetic variation of the genotyped HELIUS cohort as implemented in PLINK (3). For the analysis the same quality-controlled was used as descripted in the methods for the EIGENSTRAT analysis (4). Analysis were performed with inclusion of all 1000 genomes European populations including the Dutch and Turkish HELIUS individuals and a second one with also inclusion of the Moroccan individuals.

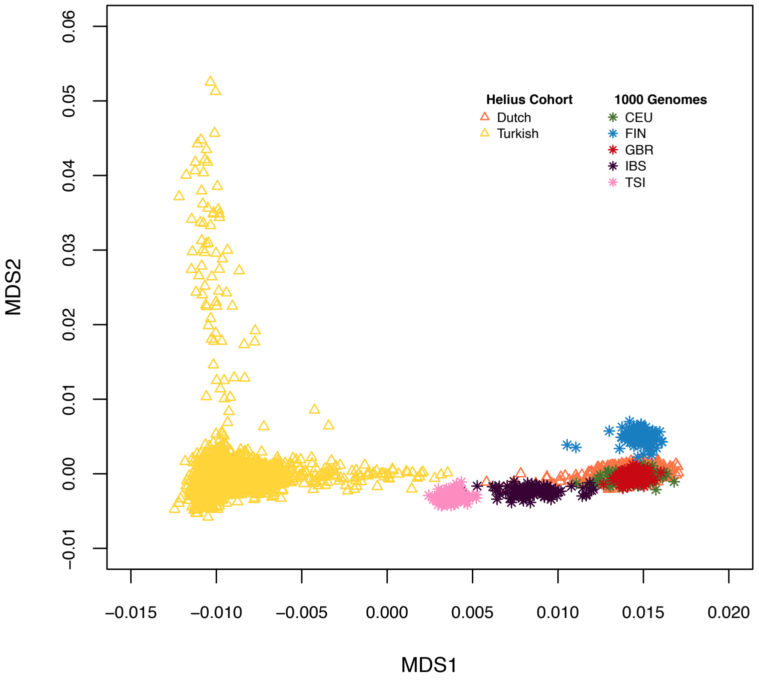

A). Displaying MDS results zooming in on the 1000 Genome European populations combined with the Dutch and Turkish HELIUS cohort individuals. The first two multidimensional scales were plotted against each other. A European South to North gradient was observed. Notable is the structure found within the Turkish individuals. This structure could be the result of the South-eastern Europe and Western Asia location and associated history.

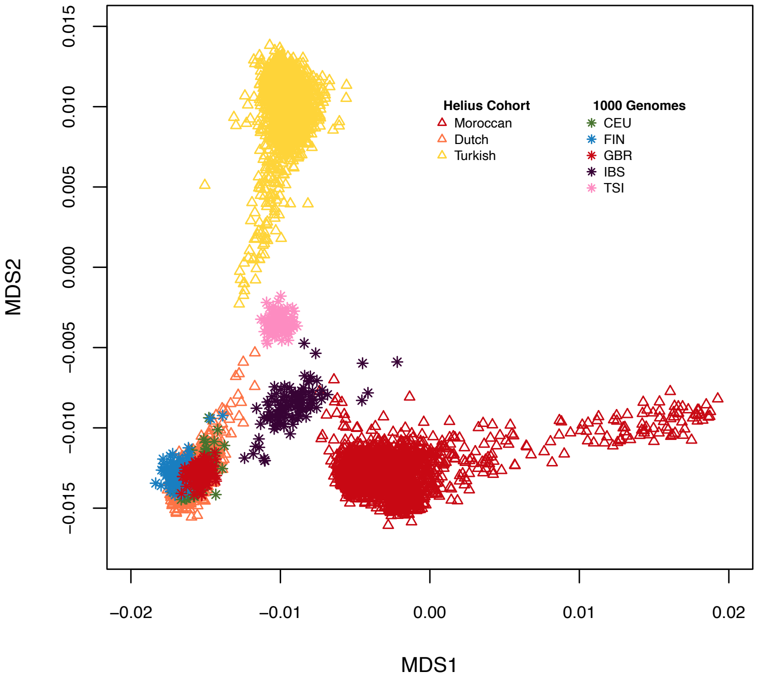

B). Displays the results when repeating the MDS analysis with the inclusion of the Moroccan individuals. Within the Moroccan group a substructure is seen towards the African ancestry, as seen in figure 1A of the manuscript (5).

Overall, the MDS analyses showed that how more you zoom in to specific closely related populations you define more structure within and between the included samples. Also it shows the importance of correcting for the relatedness and population structures when conducting association studies including samples from multiple ethnicities.

Figure S3

Ancestry estimated using ADMIXTURE for HELIUS samples including the 1000 genomes (6). Header of the figure displays full names of all the HELIUS cohort populations. Boxes above the figure include the 5 super population abbreviations used by 1000 genomes. Numbers represent the 1000 genome populations in the order they are included in the legend below. Cross validation (CV) for each K is included in the plot shown within the legend. The CV flattening at a certain K, which stands for the number of ancestral populations, gives the best estimate.
Next page displays the ADMIXTURE results for 2 up till 10 source populations. Different colours by each K, indicated with the number, corresponds with the total amount of source populations. Colours within each K plot were given randomly and similar colours between plots have no meaning.

African (AFR)
1. YRI Yoruba in Ibadan, Nigeria
2. MSL Mende in Sierra Leone
3. ESN Esan in Nigeria
4. LWK Luhya in Webuye, Kenya
5. GWD Gambian in Western Divisions in the Gambia
6. ACB African Caribbeans in Barbados
7. ASW Americans of African Ancestry in SW USA

European (EUR)
8. CEU Utah Residents (CEPH) with Northern and Western European Ancestry
9. GBR British in England and Scotland
10. IBS Iberian Population in Spain
11. TSI Toscani in Italia
12. FIN Finnish in Finland

South Asian (SAS)
13. GIH Gujarati Indian from Houston, Texas
14. PJL Punjabi from Lahore, Pakistan
15. STU Sri Lankan Tamil from the UK
16. ITU Indian Telugu from the UK
17. BEB Bengali from Bangladesh

East Asian (EAS)
18. CHB Han Chinese in Beijing, China
19. JPT Japanese in Tokyo, Japan
20. CHS Southern Han Chinese
21. CDX Chinese Dai in Xishuangbanna, China
22. KHV Kinh in Ho Chi Minh City, Vietnam

Ad Mixed American (AMR)
23. PEL Peruvians from Lima, Peru
24. MXL Mexican Ancestry from Los Angeles USA
25. PUR Puerto Ricans from Puerto Rico
26. CLM Colombians from Medellin, Colombia

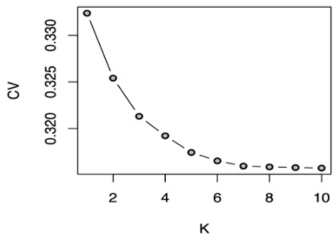

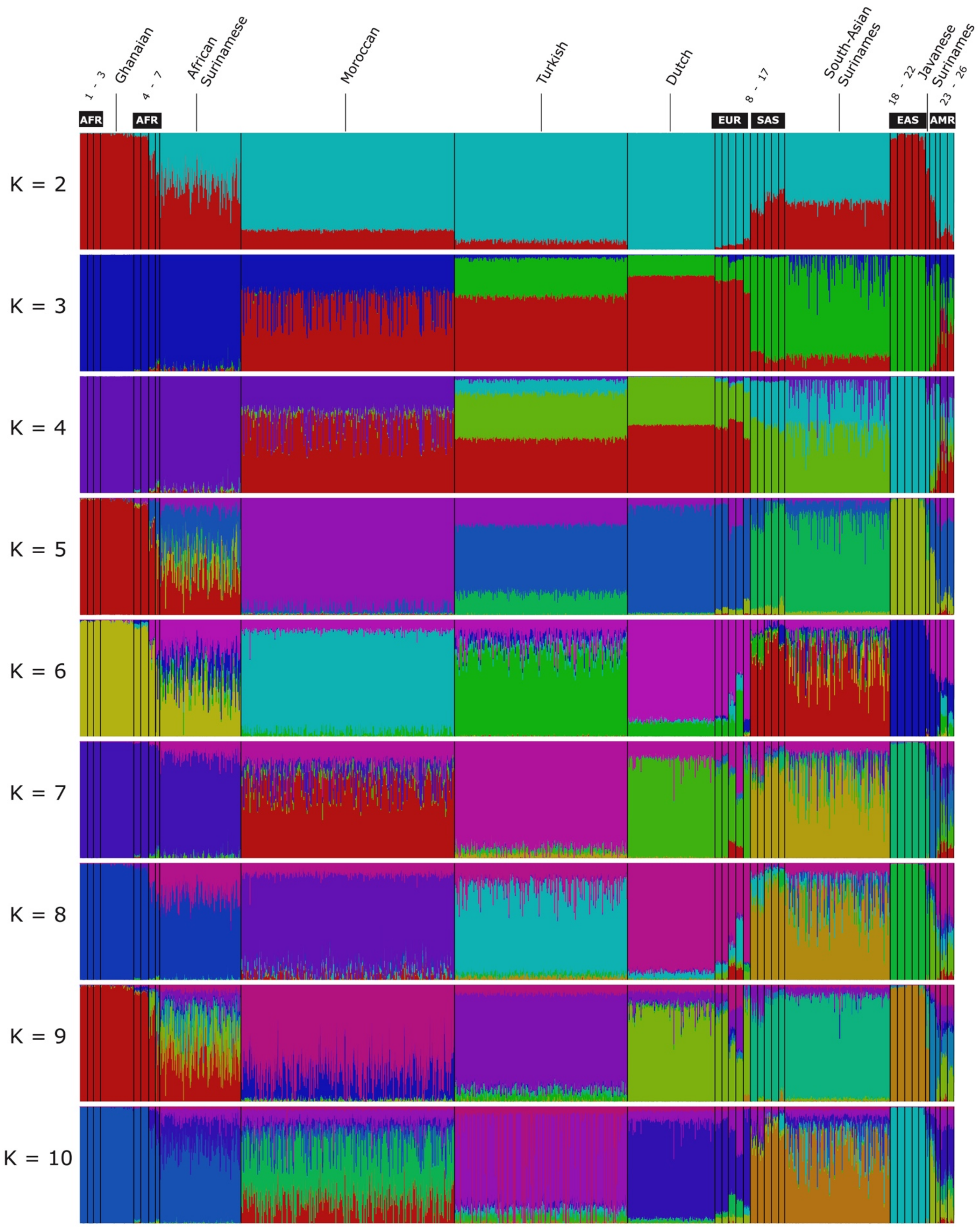

1 - 3
Ghanaian
4 - 7
African Surinamese
Moroccan
Turkish
Dutch
8 - 17
South-Asian Surinames
18 - 22
Javanese Surinames
23 - 26
AFR
AFR
EUR
SAS
EAS
AMR
K = 2
K = 3
K = 4
K = 5
K = 6
K = 7
K = 8
K = 9
K = 10

Figure S4

Meta-analyisis of the GWAS results using different methods. Compared were the fixed-effect (FE), random-effect (SE), a Han and Eskin's Random Effects model (SE2), MR-MEGA meta-analysis and the mixed linear model on all samples together named Joint (7,8). Each axis represents the -log10P of one of the methodologies plotted against one other method. Diagonal line indicates that observed p-values between both methods are equal.

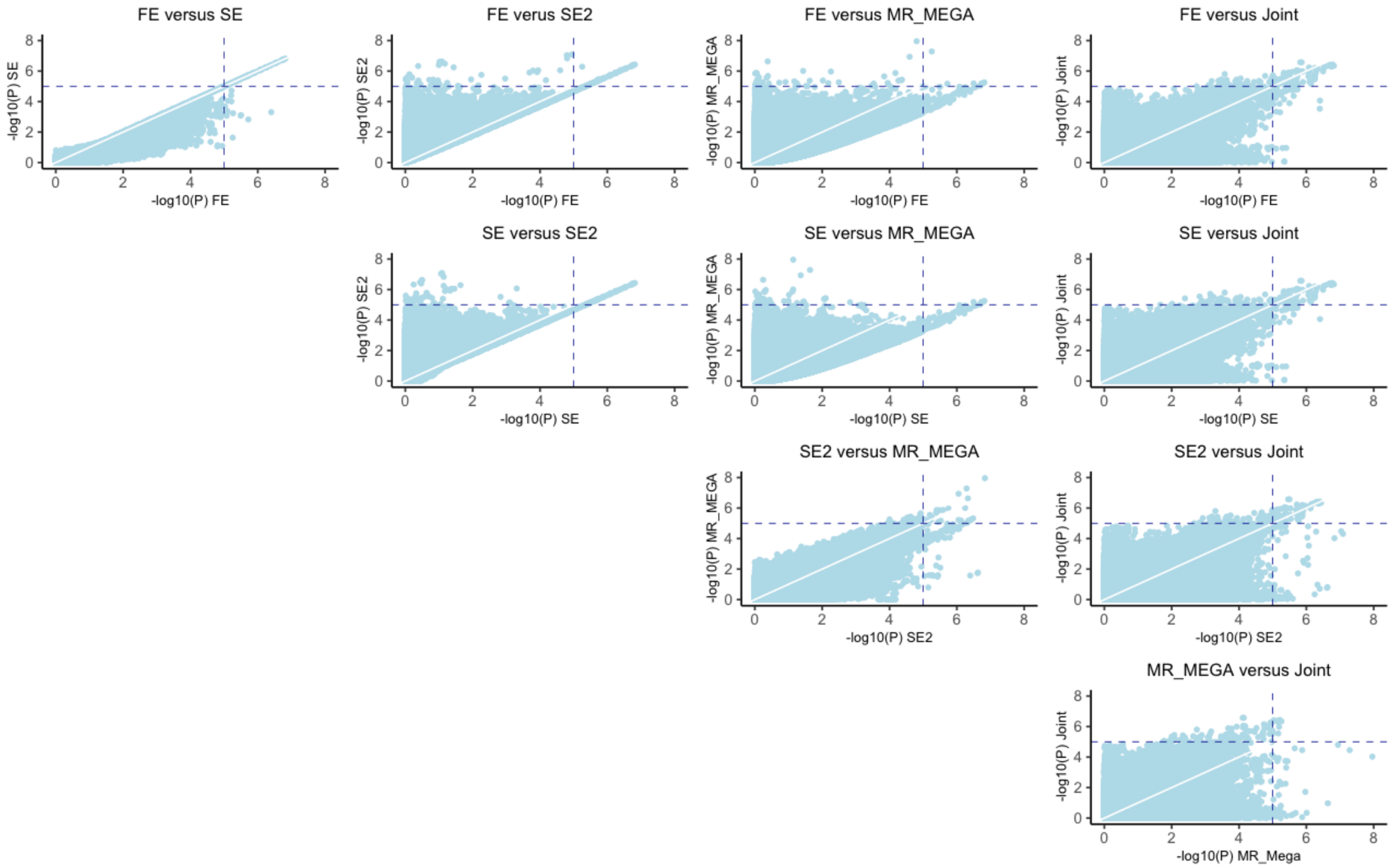
FE versus SE
-log10(P) SE
-log10(P) FE
FE verus SE2
-log10(P) SE2
-log10(P) FE
FE versus MR_MEGA
-log10(P) MR_MEGA
-log10(P) FE
FE versus Joint
-log10(P) Joint
-log10(P) FE
SE versus SE2
-log10(P) SE2
-log10(P) SE
SE versus MR_MEGA
-log10(P) MR_MEGA
-log10(P) SE
SE versus Joint
-log10(P) Joint
-log10(P) SE
SE2 versus MR_MEGA
-log10(P) MR_MEGA
-log10(P) SE2
SE2 versus Joint
-log10(P) Joint
-log10(P) SE2
MR_MEGA versus Joint
-log10(P) Joint
-log10(P) MR_Mega

<u>Figure S5</u>

A). Miami plot showing the meta-analysis results from the MANTRA and MR-Mega meta-analysis (8,9). Most meta-analysis were developed and used on European decent populations. Several specific trans-ethnic meta-analysis methods have been developed. Two of these are MR-Mega and the Bayesian MANTRA. Both methods have been used to perform a meta-analysis on the individual ethnicity GWAS results from the HELIUS cohort. On the y axis are the -log10P values for MR-Mega on the lower part and the positive log10Bayes factor in favor of association in the upper part. Results per chromosome for all tests are displayed at the x-axis. Strongest associations are marked with a letter (the same regions between both methods have the same overlapping letter). Both methods showed overlap in the association signals of rs57061241 (A), rs9407060 (E) and rs17175602 (F). The association of markers B_1 and B_2 represent the same signal and both markers were in complete LD. For a translation of all letters to rs numbers see figure S5B.

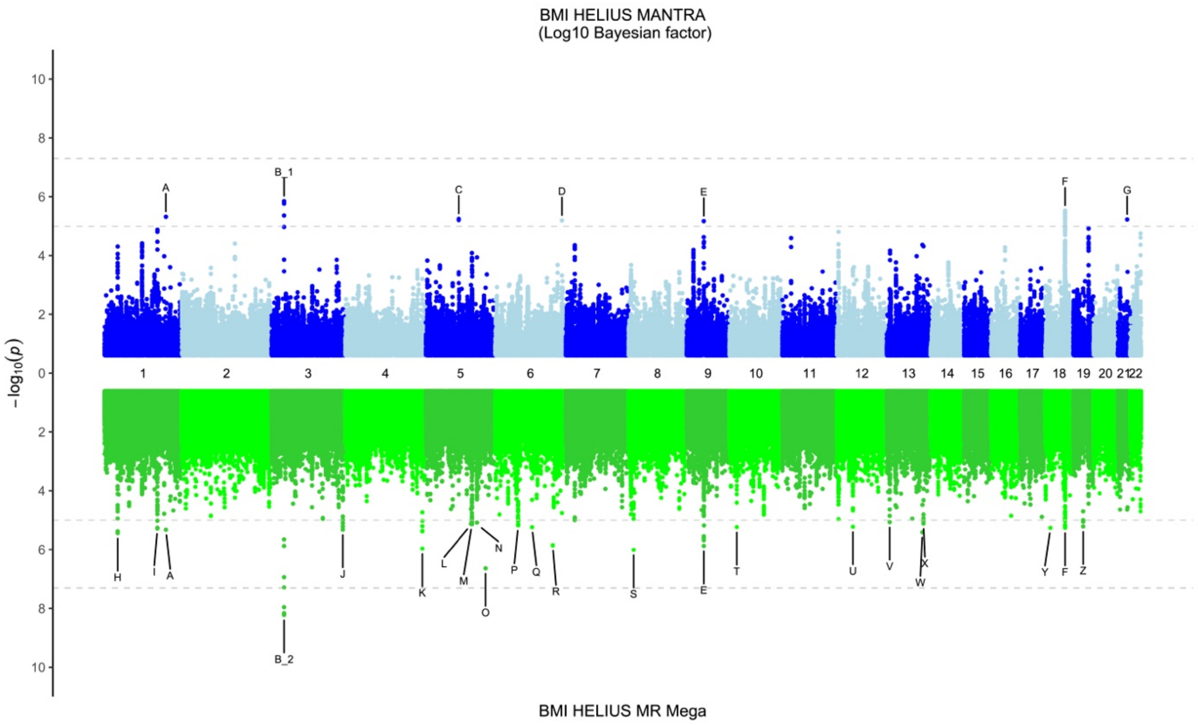

B). Allele frequencies of the strongest associated markers from MR-Mega and MANTRA meta-analysis. Y-axis shows the frequency of the alleles and x-axis displays all markers of the Miami plot shown in A) indicated with the letter and followed by the rs number.

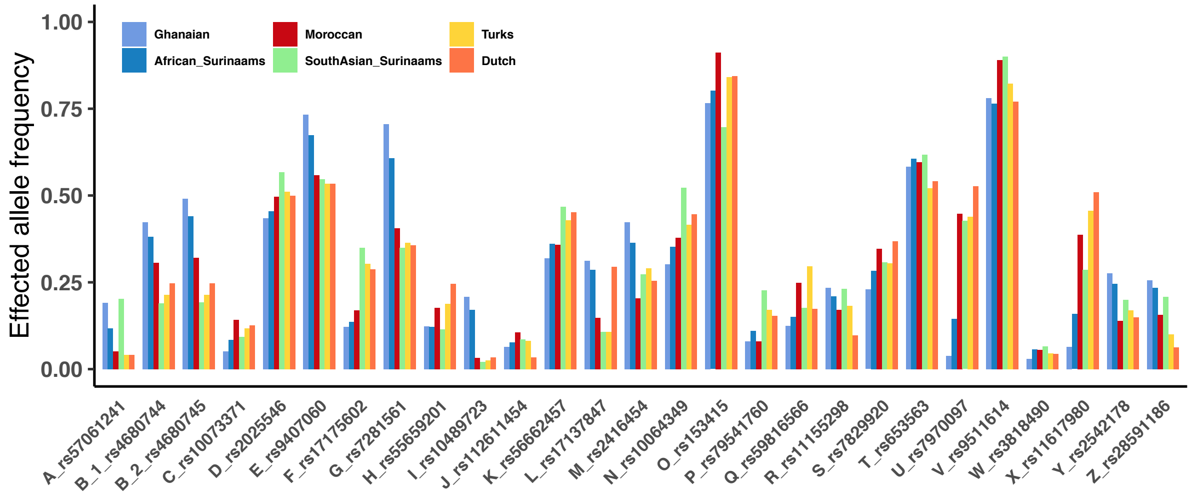

References

1. A. E. Locke *et al.*, Genetic studies of body mass index yield new insights for obesity biology. *Nature* **518**, 197-206 (2015).
2. M. C. Y. Ng *et al.*, Discovery and fine-mapping of adiposity loci using high density imputation of genome-wide association studies in individuals of African ancestry: African Ancestry Anthropometry Genetics Consortium. *PLoS Genet* **13**, e1006719 (2017).
3. S. Purcell *et al.*, PLINK: a tool set for whole-genome association and population-based linkage analyses. *Am J Hum Genet* **81**, 559-575 (2007).
4. N. Patterson, A. L. Price, D. Reich, Population structure and eigenanalysis. *PLoS Genet* **2**, e190 (2006).
5. B. M. Henn *et al.*, Genomic ancestry of North Africans supports back-to-Africa migrations. *PLoS Genet* **8**, e1002397 (2012).
6. D. H. Alexander, K. Lange, Enhancements to the ADMIXTURE algorithm for individual ancestry estimation. *BMC Bioinformatics* **12**, 246 (2011).
7. Han, E. Eskin, Random-effects model aimed at discovering associations in meta-analysis of genome-wide association studies. *Am J Hum Genet* **88**, 586-598 (2011)
8. R. Magi *et al.*, Trans-ethnic meta-regression of genome-wide association studies accounting for ancestry increases power for discovery and improves fine-mapping resolution. *Hum Mol Genet* **26**, 3639-3650 (2017).
9. A. P. Morris, Transethnic meta-analysis of genomewide association studies. *Genet Epidemiol* **35**, 809-822 (2011).
